# Network Structure of Comorbidity Patterns in U.S. Adults with Depression: A National Study Based on Data from the Behavioral Risk Factor Surveillance System

**DOI:** 10.1155/2023/9969532

**Published:** 2023-04-15

**Authors:** Cristian Ramos-Vera, Antonio Serpa Barrientos, José Vallejos-Saldarriaga, Yaquelin E. Calizaya-Milla, Jacksaint Saintila

**Affiliations:** ^1^Research Area, Faculty of Health Sciences, Universidad César Vallejo, Lima, Peru; ^2^Faculty of Psychology, Universidad Nacional Mayor de San Marcos, Lima, Peru; ^3^Research Group for Nutrition and Lifestyle, School of Human Nutrition, Universidad Peruana Unión, Lima, Peru

## Abstract

**Background:**

People with depression are at increased risk for comorbidities; however, the clustering of comorbidity patterns in these patients is still unclear.

**Objective:**

The aim of the study was to identify latent comorbidity patterns and explore the comorbidity network structure that included 12 chronic conditions in adults diagnosed with depressive disorder.

**Methods:**

A cross-sectional study was conducted based on secondary data from the 2017 behavioral risk factor surveillance system (BRFSS) covering all 50 American states. A sample of 89,209 U.S. participants, 29,079 men and 60,063 women aged 18 years or older, was considered using exploratory graphical analysis (EGA), a statistical graphical model that includes algorithms for grouping and factoring variables in a multivariate system of network relationships.

**Results:**

The EGA findings show that the network presents 3 latent comorbidity patterns, i.e., that comorbidities are grouped into 3 factors. The first group was composed of 7 comorbidities (obesity, cancer, high blood pressure, high blood cholesterol, arthritis, kidney disease, and diabetes). The second pattern of latent comorbidity included the diagnosis of asthma and respiratory diseases. The last factor grouped 3 conditions (heart attack, coronary heart disease, and stroke). Hypertension reported higher measures of network centrality.

**Conclusion:**

Associations between chronic conditions were reported; furthermore, they were grouped into 3 latent dimensions of comorbidity and reported network factor loadings. The implementation of care and treatment guidelines and protocols for patients with depressive symptomatology and multimorbidity is suggested.

## 1. Introduction

Depression represents a major problem for public health systems [1] and is defined as a debilitated mood state characterized by loss of positive affect and a variety of emotional, cognitive, and behavioral symptoms, such as anhedonia, inappropriate sleep and eating behaviors, worthlessness, and recurrent thoughts of death [2]. In particular, depression can become a serious health condition when it is recurrent and of moderate or severe intensity [3]. According to the World Health Organization (WHO), an estimated 300 million people worldwide suffer from this mental disorder [1]. In the Latin America and Caribbean region, depression is the leading cause of disability, affecting 7.8% of the population, especially among the 15-50 age group [4]. In the United States (U.S.A), during the COVID-19 pandemic, symptoms of depression tripled during the early 2020 in U.S. adults, from 8.5% before the pandemic to a staggering 27.8% [5]. In fact, depression even worsened in 2021, reaching 32.8%, which equates to 1 in 3 U.S. adults [5].

On the other hand, in the United States, there are public health challenges related to chronic diseases, which pose a threat to the social economy in the country. In 2018, a cross-sectional survey conducted by the US National Center for Health Statistics (NCHS) that included 25,417 adults found that 51.8% were living with at least 1 of 10 chronic conditions, while 27.2% had multiple chronic conditions [6].

Likewise, depression is usually present in many chronic diseases, such as cancer [7], cardiovascular problems, type 2 diabetes [8], obesity [9], and other metabolic and neurological conditions [10]. The prevalence of depression in these patients is often significantly higher compared to the general population and represents a substantial part of the psychosocial burden of these diseases [10]. Making a definitive diagnosis of depression in patients with chronic comorbidities can be particularly difficult due to overlapping clinical symptoms [11]. Undetected depression in primary care represents a serious concern considering the burden associated with chronic conditions [12]. The factors that contribute to the onset of depression in patients with chronic diseases are multiple, among them the following stand out: shared psychological, social, and genetic factors; convergent biological pathways; and health behaviors [10]. Therefore, the approach to depression among patients with chronic diseases should be made considering several associated factors. For this, it is necessary that specialized hospital centers have specialists trained and equipped to recognize depression among these patients.

Furthermore, suffering from a chronic condition can affect a person's overall well-being [2]. People affected by chronic diseases are more prone to a higher risk of mental illness and a poorer quality of life [13]. Especially people with chronic conditions are at greater risk of experiencing depressive symptoms [14, 15]. Previous studies have documented associations between chronic conditions and depressive symptoms in American adults [15]. In fact, available evidence reports that depression occurs in 7% to 50% of cancer patients [16] and 5%-54% of patients in the acute poststroke period [17]. Similarly, depression increases the prevalence of death among patients with chronic heart failure and heart transplantation [18, 19]. Between 30% and 45% of patients with coronary artery disease report depressive symptoms in clinical settings [20]. Moreover, studies reveal that between 5.8% and 43.3% of patients with diabetes suffer from depression [21], while among patients with chronic obstructive pulmonary disease (COPD), the prevalence rate of depression is from 8% to 80% [18].

It should be recognized that mental health diagnoses have been recognized as an important component of multimorbidity in patients [22]; however, there are few studies in adults with depression that have documented the network structure of chronic comorbidity. In fact, there is a lack of evidence regarding the clustering of physical comorbidities in individuals with depression [23]. The paucity of studies, the increasing prevalence of comorbid illness, and the burden of depression in adults evidence the need to identify latent comorbidity patterns and explore the network structure of chronic comorbidity. A detailed and accurate understanding of chronic comorbidities and depression is essential for public health organizations and agencies because it will enable the design of policies and the implementation of policies and strategies for the effective and dynamic prevention and control of these disorders.

The present study used an approach based on a multivariate graphical network model and factor analysis (exploratory graphical analysis) to identify patterns of latent comorbidity in adults who were diagnosed with a depressive disorder. We hope that this novel systematic analysis will improve the understanding of how most comorbid physical diseases and their underlying covariance interact. Therefore, there is a need to investigate the network structure of chronic comorbidity in participants suffering from such a psychiatric condition with the highest prevalence in the USA.

## 2. Materials and Methods

### 2.1. Data Source

This study used data from the 2017 Behavioral Risk Factor Surveillance System (BRFSS). This study analyzed 89,209 of 450,013 U.S. adults aged 18 years and older. The study sample consisted of 29,079 men and 60,063 women who presented with depression. The BRFSS was conducted through a telephone survey using a random-digit-dialing technique for both landline and cell phones to collect statewide data on U.S. residents regarding their health-related risk behaviors, chronic health conditions, and use of preventive services (response rate: landline phone (45.3%) and cell phone (44.5%)) [24]. The BRFSS conducts an annual survey of over 400,000 adults, making it the most extensive health survey system in the world that is conducted regularly [24]. The BRFSS data offers insights into health-risk behaviors, chronic health issues, and sociodemographic characteristics in metropolitan areas and adjacent counties across the United States [24, 25].

### 2.2. Depression

To determine the presence of depression, the following question was asked: has a doctor, nurse, or other health professionals ever told you that you have a depressive disorder, including depression, major depression, dysthymia, or minor depression? Response items were dichotomized as follows: yes or no. Prior research that has utilized BRFSS data has employed a comparable definition to detect the existence of depression [26–28].

### 2.3. Chronic Conditions

Data on chronic diseases were collected by asking participants if they suffered from any of the following diseases: diabetes or high blood sugar, hypertension or high blood pressure, obesity, cancer, high blood cholesterol, myocardial infarction, coronary heart disease, stroke, asthma, respiratory diseases, arthritis, and kidney disease. Subsequently, the responses were then dichotomized as follows: yes or no [25].

### 2.4. Sociodemographic Data

Sociodemographic characteristics were considered, such as age in years (18-34, 35-44, 45-54, 55-64, and 65+), marital status (living as a couple, previously married, and single), education (≤ high school, ≥ bachelor's degree, and college and technical school), employment (yes or no), range of income (<$25,000, $25,000-$74,999, and ≥$75,000), and race (White non-Hispanic, African American, Asian, Hispanic, and other).

### 2.5. Statistical Analysis

For the analyses, we have considered exploratory graphical analysis (EGA), through the triangulated maximally filtered graph (TMFG) or EGAtmfg, where correlations are structurally adjusted [25, 29, 30]. The four variables with the highest partial correlations were summed one by one iteratively after being connected; based on this, the sum of the highest partial correlations of the three variables whose nodes are included in the network was run [25, 31, 32]. This process allowed a cluster analysis to be performed using the Leiden algorithm, which is a modification of the Louvain algorithm designed to detect communities with greater speed and accuracy [25, 33]. In the network, it can be seen that the strength of the connection represents the intensity of the unique association between two variables.

The “EGAnet” package available in the R environment was used [30, 34]. It is worth mentioning that the network method uses the TMFG procedure including a cluster selection and using the Leiden/Louvain algorithm, which is considered as an option in the “EGAnet” package. Subsequently, the graphical model, edge weights, and clusters of chronic conditions were analyzed. The “EGA.estimate” function was used to obtain the weight matrix. After this result, the “bootEGA” function was used to obtain the estimated network structure based on the bootstrap method, using the same parameters with 200 permutations. The “cor_auto” argument, constituted by Lavaan's lavCor function, was taken into account for the analysis of both models. [35], which allowed the calculation of a correlation matrix based on Pearson's correlation. Through this analysis, all factors are eliminated by looking at possible ordinal variables or variables that constitute a maximum of 7 unique integer values. Additionally, a check was performed to explore whether the correlation matrix is positively definite [36].

After obtaining the bootstrap model, the factor loadings, known as standardized node strengths, were explored; the “net.loads” function was used for this purpose. We have considered the “dimensionStability” function to analyze the structural consistency of the predicted network model and the stability of the extracted clusters. For the individual examination of the consistency of the elements in the clusters, we have used the “itemStability*”* function [25].

Furthermore, using the node strength centrality metric, the strength with which a node is directly connected to other nodes, based on the absolute sum of the edge weights, we identified which nodes are most important to the network, using the “centralityPlot” function of the R package qgraph [25]. The EGA is a novel method compared to traditional factor analyses such as parallel analysis or the Kaiser criterion; their use is particularly important in models with unbalanced structures, low factor loadings, binary data, and large sample sizes such as those used in the present study [37, 38].

Lastly, the “entropyFit” and “tefi” functions: entropy fit index with von Neumann entropy (EFI.vn) and total EFI with von Neumann entropy (TEFI.vn) were used to analyze two entropic fit indexes. For this purpose, in addition to using Shannon's entropy [39], quantum information metrics were also used, through the correlation matrix [40]. It is worth mentioning that these values are more accurate when used to identify latent factors. Values close to zero of EFI indicate that there is less disorder (or lack of certainty) in the variable system [25, 30].

## 3. Results

The largest proportion of participants was over 55 years of age (49.1%), and 61.5% were married. 40.2% and 79.8% reported monthly incomes of $25,000-$74,999 and were non-Hispanic White, respectively (Table 1).

**Figure 1:**
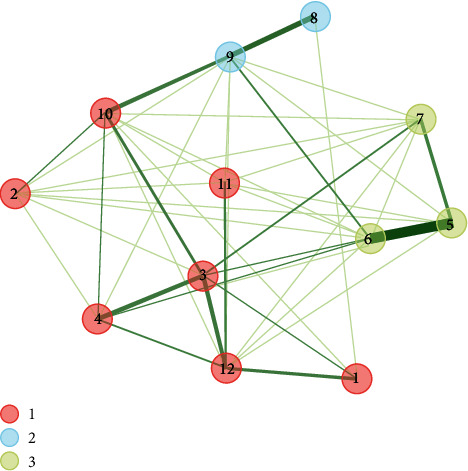
EGA of comorbidity patterns. Note: the greater the intensity of the connection, the greater the positive association. The thickness of the line is equivalent to the magnitude of the ratio. (1) Obesity, (2) cancer, (3) high blood pressure, (4) high blood cholesterol, (5) heart attack, (6) coronary heart disease, (7) stroke, (8) asthma, (9) respiratory diseases, (10) arthritis, (11) kidney disease, and (12) diabetes.

The findings of the EGA demonstrate that the network exhibits three latent comorbidity patterns, wherein chronic illnesses are categorized into three factors (as depicted in Figure 1). A cluster analysis was considered, which was detected with the Leiden algorithm, which is a modification of the Louvain algorithm for community detection, which refers to greater precision in the groupings of network systems with cross-sectional data. [30, 34] The first group was composed of 7 comorbidities (obesity, cancer, high blood pressure, high blood cholesterol, arthritis, kidney disease, and diabetes). The second pattern of latent comorbidity included the diagnosis of asthma and respiratory diseases. The last factor grouped 3 conditions (heart attack, coronary heart disease, and stroke).

The EGA, in addition to specifying the graphical model and the clusters, reports the weights of the edges (partial relationships), which can be seen in a correlation matrix based on Pearson-type statistical associations. The EGA correlation matrix can be found in Supplement material 1.

**Figure 2:**
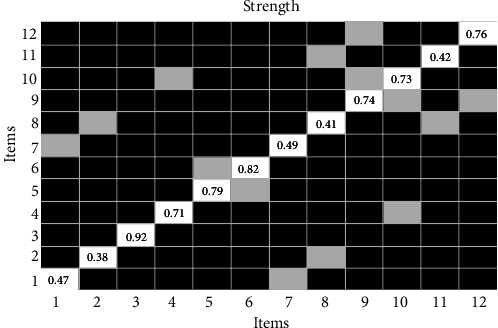
Centrality indexes of chronic conditions. Note: numbers refer to a chronic condition identified in Table 2. (1) Obesity, (2) cancer, (3) high blood pressure, (4) high blood cholesterol, (5) heart attack, (6) coronary heart disease, (7) stroke, (8) asthma, (9) respiratory diseases, (10) arthritis, (11) kidney disease, and (12) diabetes. This figure refers to the Bootstrap difference test for node strength, where gray boxes indicate nonsignificant differences and black boxes indicate significant differences.

Table 2 displays the item (comorbidities) assignments and network loads. The estimates of centrality (Figure 2) in the network show that the conditions of hypertension (item 3) and coronary heart disease (item 6) were more central in the network, that is, they presented a greater number and magnitude of connections (partial associations). However, hypertension reported a centrality value (.92) statistically different from the other chronic diseases. The conditions with lower centrality magnitudes were asthma and kidney disease.

**Figure 3:**
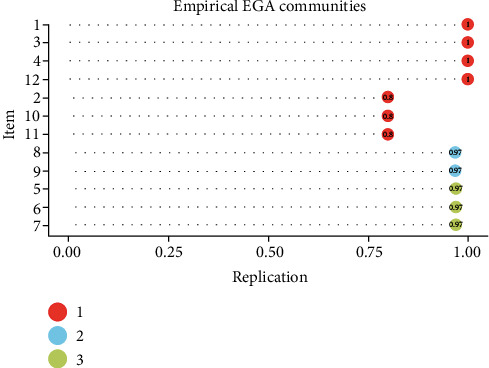
Stability of the 12 chronic conditions of the EGA. Note: in the EGA, the nodes correspond to each instance of the item (comorbidity) in the initial dimension identified by the analysis. (1) Obesity, (2) cancer, (3) high blood pressure, (4) high blood cholesterol, (5) heart attack, (6) coronary heart disease, (7) stroke, (8) asthma, (9) respiratory diseases, (10) arthritis, (11) kidney disease, and (12) diabetes.

Figure 3 shows that some comorbidities (cancer, arthritis, and kidney disease) of dimension 1 were less consistent within such a latent community given a lower prevalence in the population assessed, while the other chronic diseases identified in factors 2 and 3 refer to a higher latent strength. Network factor loadings specific to the comorbidities in the EGA can be found in Supplement material 2.

## 4. Discussion

The prevalence of depression in adult patients with chronic conditions, such as obesity, diabetes, and cardiovascular disease, among others, continues to increase [15]. The psychological and clinical approach to depressive symptomatology should be carried out taking into account factors such as noncommunicable diseases. Therefore, it is important to use nationally representative repeated cross-sectional data to examine the association between these variables. This study identified and explored the associations and coexistence of chronic comorbidities using a network structure in 89,209 adults, a national sample spanning all 50 American states who suffered from depression, one of the most prevalent psychiatric conditions in the USA [5]. These findings serve to provide significant information to health professionals, particularly physicians.

The findings of the current study identified three comorbidity patterns. The first factor was composed of obesity-hypertension-high cholesterol-diabetes-cancer-arthritis-kidney diseases. Other investigations of comorbidity networks in national samples from Europe and Asia have reported clusters of arthritis with metabolic diseases [41, 42], while other nationwide longitudinal cohort studies report comorbidity patterns that group cancer and kidney disease with such metabolic conditions [43, 44]. The second comorbid dimension integrated asthma-respiratory disease, and the third latent group jointly identified cardiovascular disease (heart attack-coronary heart disease-stroke conditions). The latter two factors have been reported most frequently in various studies of comorbidity patterns using factor analysis and multivariate relationship network models [45, 46].

Latent network burdens related to arthritis and coronary heart disease have also been identified that share comorbid dimensions with asthma and respiratory disease, suggesting that there may be an underlying connection between these conditions and their relationship with depression. In fact, it has been reported in another study of 74220 hospitalized patients in Spain that such characteristics cluster in a community of chronic comorbidities [47]. Another investigation that included adults with mental disorders (e.g., depression) reported a comorbidity group with a higher prevalence of heart disease, respiratory disease, arthritis, kidney disease, and hypertension [23]. The present study reports that these chronic conditions present cross-network loadings on all three factors and are associated to a greater degree, indicating certain comorbidity pathways in the systemic representation of multimorbidity in American adults with depression.

A greater centrality of hypertension refers to its greater influence on connections and network structure. In addition to its higher prevalence in the American population with depression, it shares greater associations and network load with cardiac conditions. Heart disease is a global health problem, and high blood pressure is a risk factor for most cardiovascular diseases acquired during life, such as coronary heart disease, left ventricular hypertrophy, valvular disease, arrhythmias such as atrial fibrillation, and stroke [48, 49]. The continuing relationship between blood pressure and cardiac events and the pathophysiological and biological pathways of blood pressure as one of the risk factors are complex; nonetheless, there are proposed mechanisms, including the impact of blood pressure as a physical force in the formation of atherosclerotic plaques and the association between pulsatile hemodynamics/arterial stiffness and the blood supply to the coronary arteries [50]. Likewise, depressive symptomatology constitutes one of the risk factors for mortality among patients with cardiac and hypertensive conditions [51]. In fact, scientific evidence suggests that the association between hypertension and cardiovascular disease mortality is strongest when combined with the most common mental disorders, such as depression and anxiety [52]. This evidence could explain the role of hypertension in chronic multimorbidity in people with depressive disorders.

### 4.1. Multimorbidity Patterns

#### 4.1.1. Obesity-Hypertension-High Cholesterol-Diabetes-Cancer-Arthritis-Kidney Diseases

Depression may coexist with metabolic comorbidities or metabolic syndrome. Metabolic syndrome, characterized by the presence of obesity, high blood pressure, high cholesterol, and diabetes, is a chronic condition prevalent among people suffering from depression [53]. These comorbidities may contribute to poor outcomes and inadequate response to treatment for depression [54]. There is some evidence to suggest that metabolic syndrome is a risk factor for developing depression [53]. A study using data from the third National Health and Nutrition Examination Survey (NHANES III) in the U.S. adult population showed that women with a history of major depression were more likely to have metabolic syndrome [55]. Particularly, findings from another study using NHANES 1999-2004 and 2005-2016 data showed that mental health disorders, among which depression, were associated with diabetes [56]. Clinical care for people with metabolic syndrome should be based not only on the therapy of physical conditions but also on mental health.

There are some mechanisms that could explain the presence of the metabolic syndrome in patients with depression. For example, the existing connections between depression and proinflammatory states, as a possible pathophysiological basis of the metabolic syndrome, could justify the presence of these factors. The presence of depression, as an inflammatory state, is detrimental and may affect the course or even the outcome of the management of metabolic syndrome, particularly diabetes and obesity [57]. Previous studies have shown that patients with depression had higher plasma levels of interleukin-6 compared to healthy controls [58]. Depression is associated with inflammation by increasing C-reactive protein (CRP) and cytokines, such as interleukin-6 (IL-6) and tumor necrosis factor-*α* (TNF-*α*) [58]. In addition, depression inhibits brain-derived neurotrophic factor (BDNF), one of the factors associated with neuronal resilience [59]. The presence of depressive symptomatology increases inflammation and decreases BDNF [58]. These consequences give depression the capacity to cause or even worsen comorbid illnesses [54]. Another possible mechanism is that, in general, depressed people tend to engage in unhealthy behaviors, such as smoking, unhealthy dietary choices, alcohol consumption, sedentary lifestyles, and nonadherence to medical treatment [60–63]. These behaviors, evidently, can lead to the development of the metabolic syndrome, although it is unlikely that unhealthy behaviors alone explain the entire relationship between depression and metabolic conditions [55].

Moreover, the first pattern of comorbidities grouped with chronic conditions such as cancer-arthritis-kidney diseases. Existing evidence demonstrates the coexistence of cancer, arthritis, and kidney diseases [64, 65]. Kidney disease represents a state of chronic inflammation arising from an increase in proinflammatory mediators, such as TNF-*α*, CRP, and interleukin-6 [66]. While anti-TNF-*α* drugs in the progression of kidney disease may favor deterioration of renal function, and their efficacy and safety have been demonstrated in cases of arthritis, however, it may contribute to an increased risk of lymphoma and skin cancer [64]. In fact, there is strong evidence that anti-TNF-alpha therapy can lead to cases of lymphomas and various malignancies [65]. Previous studies have reported malignancies induced by various anti-TNF-alpha drugs [67]. On the other hand, particularly, patients with arthritis have a significantly high risk of kidney disease [68]. Patients with early arthritis usually present with proteinuria, hematuria, and kidney dysfunction [69]; in addition, this elevated risk could be attributed to the presence of glomerulonephritis, renal toxicity, due to the intake of antirheumatic drugs, potentially nephrotoxic [68], and chronic inflammation caused by comorbidities, the metabolic syndrome, particularly diabetes and arterial hypertension [64], which could explain the clustering of these “latent” factors (obesity-hypertension-high cholesterol-diabetes-cancer-arthritis-kidney diseases) evidenced in the factor analysis in our study. The factor analysis applied in the current study argues that these associated chronic conditions share a common underlying trait that is responsible for the correlation between them [70].

Cancer, arthritis, and kidney diseases are all linked to depression [16]. In fact, depression proves to be detrimental to the course and outcome of cancer [57]. The link between the state of depression and anxiety and cancer outcomes has been well documented, although it remains even [16, 71]. Wang et al., after identifying 51 eligible cohort studies with 2,611,907 participants with a mean follow-up period of 10.3 years, found that, specifically, the cooccurrence of depression and anxiety was linked to an elevated likelihood of developing lung, oral cavity, prostate, and skin cancers. Additionally, there was a heightened risk of mortality from specific cancers, including lung, bladder, breast, colorectal, hematopoietic system, kidney, and prostate cancers, and an increased risk of all-cause mortality among individuals with lung cancer who also experienced depression and anxiety [71]. Similarly, a study that looked at arthritis status in 25,990 adults who participated in NHANES from 2007 to 2018 found an association with major depression [72]. Likewise, depression appears to be more prevalent among patients with severe kidney disease than without [73]. Based on these analyses, it appears that depression could potentially play a causal role and have a prognostic effect on cancer, arthritis, and kidney disease, although there is a chance of reverse causation.

#### 4.1.2. Asthma-Respiratory Diseases

Asthma, along with other respiratory diseases, particularly COPD, represents a public health problem that affects millions of people globally [74]. Both conditions are associated with some degree of airway inflammation, due to the increased expression of inflammatory proteins observed in both cases [74]. Asthma and COPD coexist. On the one hand, it is documented that some patients with asthma may develop fixed airflow obstruction and COPD at older ages and among smokers [75], and, on the other hand, some patients presenting with COPD show clinical manifestations commonly seen in asthma [76]. Similarly, there is a coexistence between respiratory diseases and depression. In fact, one study found that depression accounted for a 43% increased risk of developing asthma among adults, although asthma was not associated with an increased risk of depression [77]. In the United States, findings from a cross-sectional study of 18,588 people participating in the Health and Retirement Survey 2004, showed that depressive symptomatology is common in COPD and is more likely to occur in COPD than in other common chronic diseases [78]. Depression is prevalent in patients with respiratory problems and poses a challenge to treatment and may increase the risk of undesirable outcomes [79].

#### 4.1.3. Cardiovascular Diseases (Heart Attack-Coronary Heart Disease-Stroke)

The last comorbidity pattern grouped these chronic conditions. Furthermore, in the network relationships between comorbidities of the same factor, a greater magnitude was observed between heart attack and coronary heart disease. This pattern has been previously described in previous factor analysis studies involving adults with depression [46, 80]. This comorbid pattern has reported that systemic network relationships are more closely linked to metabolic conditions [81, 82]. Previous studies have suggested associations between conditions in this pattern through factors such as medication use and biomarkers such as brain natriuretic peptide (BNP) and pro-BNP [83, 84]. Additionally, other possible biomarkers including anemia, C-reactive protein, and cystatin C have been identified [85]. Other studies that have reported similar comorbidity patterns have evidenced a high burden of mental illness [23]. In particular, previous studies have found strong correlations between cardiovascular disease and depression [86].

The results of this study reinforce the evidence of the link between depression and different physical conditions, which is mediated, at least in part, by common environmental factors such as diet and physical activity, among others [87], and converging psychological, social, and biological pathways [10]. Evidence of how different depression symptom profiles may be associated with different comorbid chronic conditions is necessary because it favors understanding these interactions to implement public health efforts to extend healthy living and improve people's quality of life [88].

### 4.2. Limitations

The study has some limitations that should be considered. First, one of the main limitations of this study is that there is a lack of representativeness because the data were collected through a telephone survey; in fact, response rates to telephone surveys have declined in countries such as Ireland and the United Kingdom in recent years [89]. Particularly in the United States, BRFSS rates have also declined [90]. Although the study may lack reliable information on the health status of the participants, the fact that the BRFSS has incorporated cell phone data and changes in weighting methods has indicated that the data are reliable and valid compared to other surveys [90]. Second, it is a cross-sectional study in nature; therefore, it does not allow causal inference of the observed associations between chronic conditions with comorbidity patterns. Third, because all chronic conditions were self-reported, therefore, recall bias is possible, with more severe diseases being more likely to be reported; consequently, the validity and reliability of the results may be imperfect, and their interpretation should be made with caution. Fourth, in the current study, clinical diagnoses of depressive symptoms made by a medical specialist based on the results of medical care were not used. Finally, the majority female composition of the sample could lead to a selection bias.

## 5. Conclusion

In this cross-sectional study, it was evident that there are associations between chronic conditions. These associations give rise to different patterns of comorbidity. Thus, patterns have been identified: obesity-hypertension-high cholesterol-diabetes-cancer-arthritis-kidney diseases, asthma-respiratory disease, and cardiovascular disease (heart attack-coronary heart disease-stroke). Moreover, the network relationships between comorbidities of the same factor that presented the greatest magnitude were heart attack with coronary heart disease, hypertension with diabetes, and asthma with respiratory diseases. Our findings suggest the design of policies and the implementation of policies and strategies for the effective and dynamic prevention and control of these disorders.

## Figures and Tables

**Table 1:** The sociodemographic traits and persistent health issues of study participants (*N* = 89209).

Characteristics	*N*	%
Age		
18-34	16422	18.4
35-44	12466	14.0
45-54	16501	18.5
55-64	17268	19.4
65+	26452	29.7
Marital status		
Live as a couple	51769	61.5
Previously married	22399	26.6
Single	10041	11.9
Education		
≤ High school	26344	29.5
≥ Bachelor's degree	37594	42.1
College, technical school	25271	28.3
Currently employed		
Yes	52021	59.0
No	36188	41.0
Income ranges		
< $25,000	19750	22.1
$25,000–$74,999	35819	40.2
≥ $75,000	33640	37.7
Ethnicity		
White non-Hispanic	71163	79.8
African American	7280	8.2
Asian	4905	5.5
Hispanic	499	5.6
Other	2870	1.0
Chronic condition		
High blood pressure	41984	16.3
High blood cholesterol	39563	15.4
Heart attack	7161	2.8
Coronary heart disease	7573	2.9
Stroke	6270	2.4
Asthma	21381	8.3
Cancer	18936	7.4
Respiratory diseases	14756	5.7
Arthritis	43748	17.0
Kidney disease	6111	2.4
Diabetes	16375	6.4
Obesity	33392	13.0

**Table 2:** Grouping of comorbidities according to the EGA.

Items	Chronic condition	Factor
1	Obesity	1
2	Cancer	1
3	High blood pressure	1
4	High blood cholesterol	1
5	Heart attack	3
6	Coronary heart disease	3
7	Stroke	3
8	Asthma	2
9	Respiratory diseases	2
10	Arthritis	1
11	Kidney disease	1
12	Diabetes	1

Note: the EGA refers to 3 patterns of latent comorbidity.

## Data Availability

All data used in this study are publicly available data from the National Cancer Institute and are available at http://hints.cancer.gov.

## Supplementary Materials

Supplementary 1 Supplementary material 1: EGA correlation matrix. Note: the relationships between comorbidities of the same factor of greater magnitude were between heart attack and coronary heart disease (partial *r* = .76), hypertension with diabetes (partial *r* = .51), asthma with respiratory diseases (partial *r* = .45). The strongest associations between comorbidities of different latent groupings were between respiratory disease and arthritis (partial *r* = .38), hypertension with coronary heart disease (partial *r* = .46), and heart attack (partial *r* = .42). Supplementary material 2: network loads of comorbidities in the EGA. Note: upon inspection of the network loadings, items 6 (coronary heart disease), 9 (respiratory diseases), and 10 (arthritis) report higher factorial complexity since they report higher factor loadings in two dimensions. It should be noted that network loadings tend to be lower than factor loadings since it uses a covariance of partial relationships; therefore, low values should not be interpreted as weak loads.
