# Supplementary material for: Network Structure of Comorbidity Patterns in U.S. Adults with Depression: A National Study Based on Data from the Behavioral Risk Factor Surveillance System
Source: Depress Res Treat. 2023 Apr 15;2023:9969532. doi: 10.1155/2023/9969532 (PMC10122603; doi:10.1155/2023/9969532)
Supplement: Supplementary 1 — Supplementary material 1: EGA correlation matrix. Note: the relationships between comorbidities of the same factor of greater magnitude were between heart attack and coronary heart disease (partial r = .76), hypertension with diabetes (partial r = .51), asthma with respiratory diseases (partial r = .45). The strongest associations between comorbidities of different latent groupings were between respiratory disease and arthritis (partial r = .38), hypertension with coronary heart disease (partial r = .46), and heart attack (partial r = .42). Supplementary material 2: network loads of comorbidities in the EGA. Note: upon inspection of the network loadings, items 6 (coronary heart disease), 9 (respiratory diseases), and 10 (arthritis) report higher factorial complexity since they report higher factor loadings in two dimensions. It should be noted that network loadings tend to be lower than factor loadings since it uses a covariance of partial relationships; therefore, low values should not be interpreted as weak loads. [file 9969532.f1.docx]

Supplementary material 1. EGA correlation matrix.

| Variable | Obesity | Cancer | High blood pressure | High blood cholesterol | Heart attack | Coronary heart disease | Stroke | Asthma | Respiratory diseases | Arthritis | Kidney disease | Diabetes |
| --- | --- | --- | --- | --- | --- | --- | --- | --- | --- | --- | --- | --- |
| Obesity | 1.00 |  |  |  |  |  |  |  |  |  |  |  |
| Cancer | -0.02 | 1.00 |  |  |  |  |  |  |  |  |  |  |
| High blood pressure | 0.29 | 0.19 | 1.00 |  |  |  |  |  |  |  |  |  |
| High blood cholesterol | 0.17 | 0.21 | 0.49 | 1.00 |  |  |  |  |  |  |  |  |
| Heart attack | 0.07 | 0.19 | 0.42 | 0.35 | 1.00 |  |  |  |  |  |  |  |
| Coronary heart disease | 0.11 | 0.21 | 0.46 | 0.40 | 0.76 | 1.00 |  |  |  |  |  |  |
| Stroke | 0.06 | 0.16 | 0.35 | 0.26 | 0.49 | 0.42 | 1.00 |  |  |  |  |  |
| Asthma | 0.14 | 0.02 | 0.07 | 0.04 | 0.10 | 0.13 | 0.13 | 1.00 |  |  |  |  |
| Respiratory diseases | 0.09 | 0.17 | 0.28 | 0.25 | 0.38 | 0.40 | 0.31 | 0.45 | 1.00 |  |  |  |
| Arthritis | 0.19 | 0.26 | 0.39 | 0.33 | 0.30 | 0.33 | 0.27 | 0.17 | 0.38 | 1.00 |  |  |
| Kidney disease | 0.09 | 0.20 | 0.33 | 0.23 | 0.30 | 0.34 | 0.27 | 0.10 | 0.24 | 0.28 | 1.00 |  |
| Diabetes | 0.36 | 0.13 | 0.51 | 0.43 | 0.35 | 0.37 | 0.28 | 0.11 | 0.24 | 0.31 | 0.35 | 1.00 |

Supplementary material 2. Network loads of comorbidities in the EGA.

| **Chronic Condition** | **F1** | **F3** | **F2** |
| --- | --- | --- | --- |
| Diabetes | ***0.380*** | 0.053 | 0.010 |
| High blood pressure | ***0.353*** | 0.154 | 0.000 |
| High blood cholesterol | ***0.256*** | 0.077 | 0.005 |
| Arthritis | ***0.228*** | 0.044 | **0.175** |
| Kidney disease | ***0.167*** | 0.100 | 0.026 |
| Obesity | ***0.147*** | 0.000 | 0.032 |
| Cancer | ***0.116*** | 0.029 | 0.013 |
| Heart attack | 0.064 | ***0.482*** | 0.061 |
| Coronary heart disease | **0.176** | ***0.371*** | 0.104 |
| Stroke | 0.108 | ***0.162*** | 0.071 |
| Asthma | 0.017 | 0.000 | ***0.308*** |
| Respiratory diseases | 0.120 | **0.161** | ***0.308*** |
